# A Case of Cerebellar Hippocampal and Basal Nuclei Transient Edema With Restricted Diffusion Syndrome With Poor Clinical Outcome

**DOI:** 10.7759/cureus.22767

**Published:** 2022-03-02

**Authors:** Yan Ming J Zhou, Meet Shah, Alla Fayngersh

**Affiliations:** 1 Internal Medicine, Rutgers University New Jersey Medical School, Newark, USA

**Keywords:** enlarged decompressive craniectomy, brain herniation, brain edema, substance use, chanter syndrome

## Abstract

Cerebellar hippocampal and basal nuclei transient edema with restricted diffusion (CHANTER) syndrome is a specific pattern of restricted diffusion in the hippocampi and cerebellum identified on brain imaging by clinicians in patients who present with altered mental status in the context of substance intoxication. These patients developed obstructive hydrocephalus a couple of days into their hospitalization that required therapy with osmotic agents and/or surgical interventions (i.e., drains and decompressive craniectomy). In prior cases published, many of the patients had good recovery. The case we present is of a woman who presented after polysubstance use and was found to have brain imaging findings supportive of CHANTER syndrome. Although she was treated with aggressive osmotic therapy and surgical interventions, she ultimately developed irreversible brain damage leading to an overall poor prognosis for recovery. Our case suggests variability in the progression of the syndrome and demonstrates the need for further studies to examine whether the substance of use and the patient’s chronic medical conditions may contribute to the degree of recovery.

## Introduction

Cerebellar hippocampal and basal nuclei transient edema with restricted diffusion (CHANTER) syndrome was first identified by researchers when they noted a consistent pattern of restricted diffusion in the bilateral hippocampi and cerebellar cortices in multiple patients who presented with similar symptoms of altered mental status in the context of substance intoxication [1]. They reported six similar cases of patients with these findings who developed obstructive hydrocephalus between zero and six days from admission [1]. Every patient received a mix of osmotic therapy and/or external ventricular drain (EVD) placements and subsequent suboccipital decompressive craniectomy.

We present a case of a 56-year-old female with a history of polysubstance use who presented to our medical center with altered mental status and was diagnosed with CHANTER syndrome.

## Case presentation

The patient is a 56-year-old female with a past medical history of diabetes, hypertension, polysubstance use disorder (history of benzodiazepine, heroin, and cocaine use), and osteoarthritis who was brought in via EMS after she was found unresponsive at home with reported alcohol and drug paraphernalia next to her. Her blood glucose and other vitals were unremarkable on the field. She was given 2 mg naloxone intranasal with minimal improvement in mental status.

On arrival, she was afebrile, normotensive, and saturating well on room air. Initial laboratory results showed a leukocytosis of 17.3 × 10^3^/uL (normal range (WBC): 4.5-11.0 × 10^3^/uL), creatinine of 2.3 mg/dL (normal range: 0.59-1.04 mg/dL) with baseline of 1.5 mg/dL, AST and ALT elevated to 625 U/L and 622 U/L (normal range: 10-40 U/L and 7-56 U/L, respectively), lactic acid of 7.6 mmol/L (normal range: 0.5-2.2 mmol/L), creatinine kinase of 345 U/L (normal range: 22-198 U/L), troponin of 0.61 ng/mL (normal range: 0-0.04 ng/mL), and alcohol level of 12 mg/dL. Initial computed tomography of the head (CTH) without contrast showed no acute intracranial pathology but had signs of vasogenic edema. When the patient was evaluated at the bedside, the patient was found to be lethargic but arousable. She was able to spontaneously move all extremities and appeared to have no focal neurological deficits. The patient was able to verbalize coherently that she had a severe headache.

**Figure 1:**
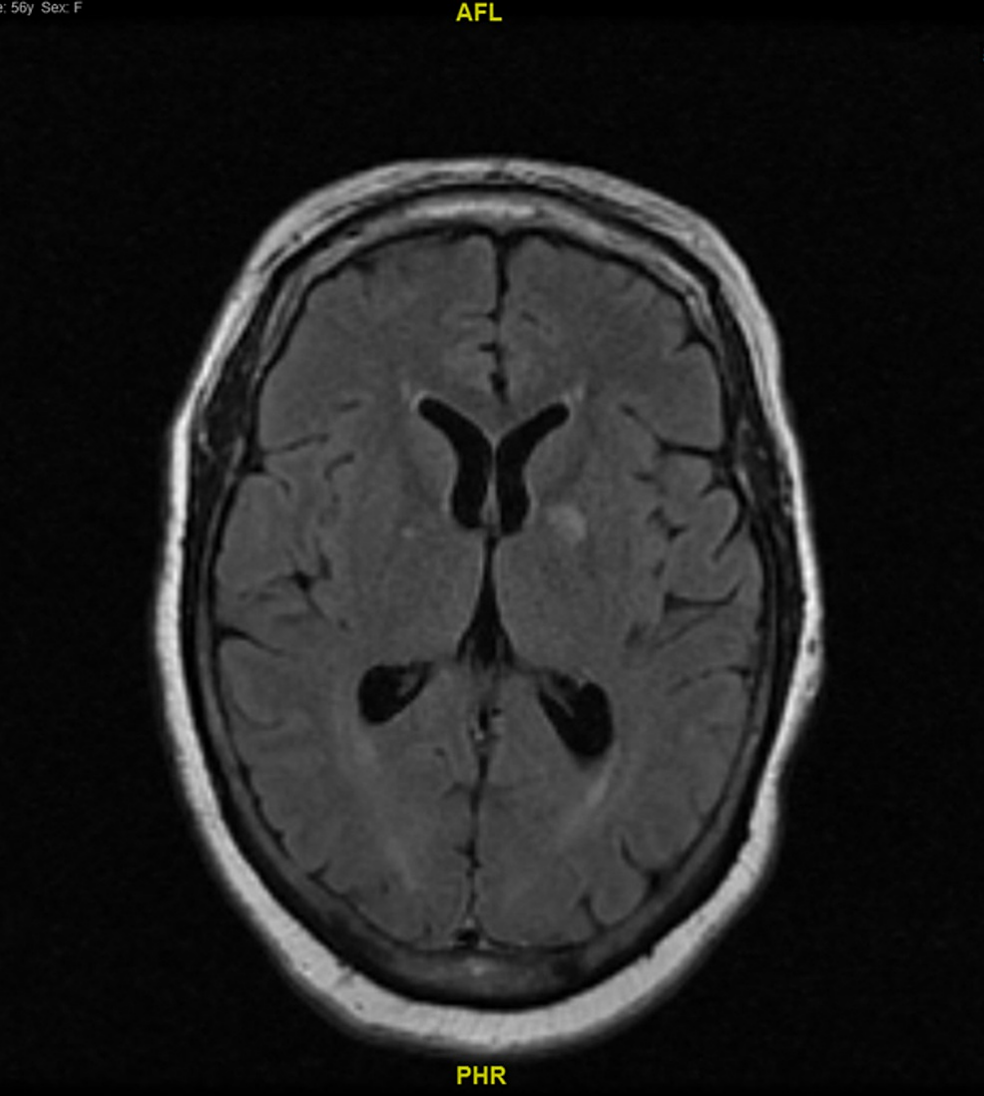
MRI of the brain without contrast in axial view on the day of admission The image shows no obstruction in the ventricles.

Given her initial CT scan findings and her mental status, a stat magnetic resonance imaging (MRI) of the brain without contrast was performed, which showed diffusion restriction in the cerebellum, hippocampus, and basal ganglia with no obstruction of ventricles (Figure 1) consistent with possible CHANTER syndrome. Post MRI, the patient became progressively more lethargic and less responsive to voice and painful stimuli. Given the patient’s deteriorating mental status post MRI, a decision was made to intubate her with subsequent bedside placement of an EVD and mannitol therapy prior to admission to the ICU. Subsequent CT angiography of the head and neck showed that she had decreased caliber of the V4 segment of bilateral vertebral arteries, irregular basilar artery likely due to mass effect, or vessel spasm but no stenosis or occlusion of the intracerebral arteries.

**Figure 2:**
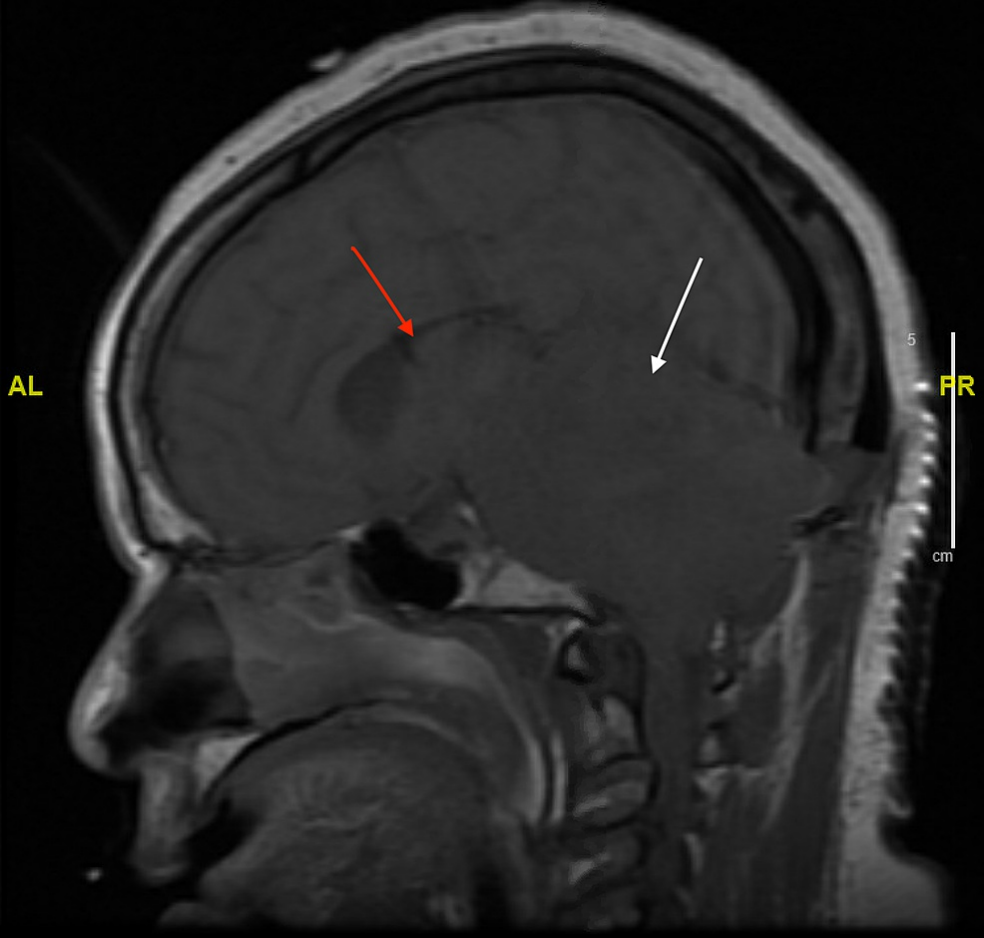
MRI of the brain without contrast in sagittal view three days into hospitalization The image shows upward and downward herniation of the cerebellar tonsils (white arrow) and mass effect on the ventricles (red arrow).

**Figure 3:**
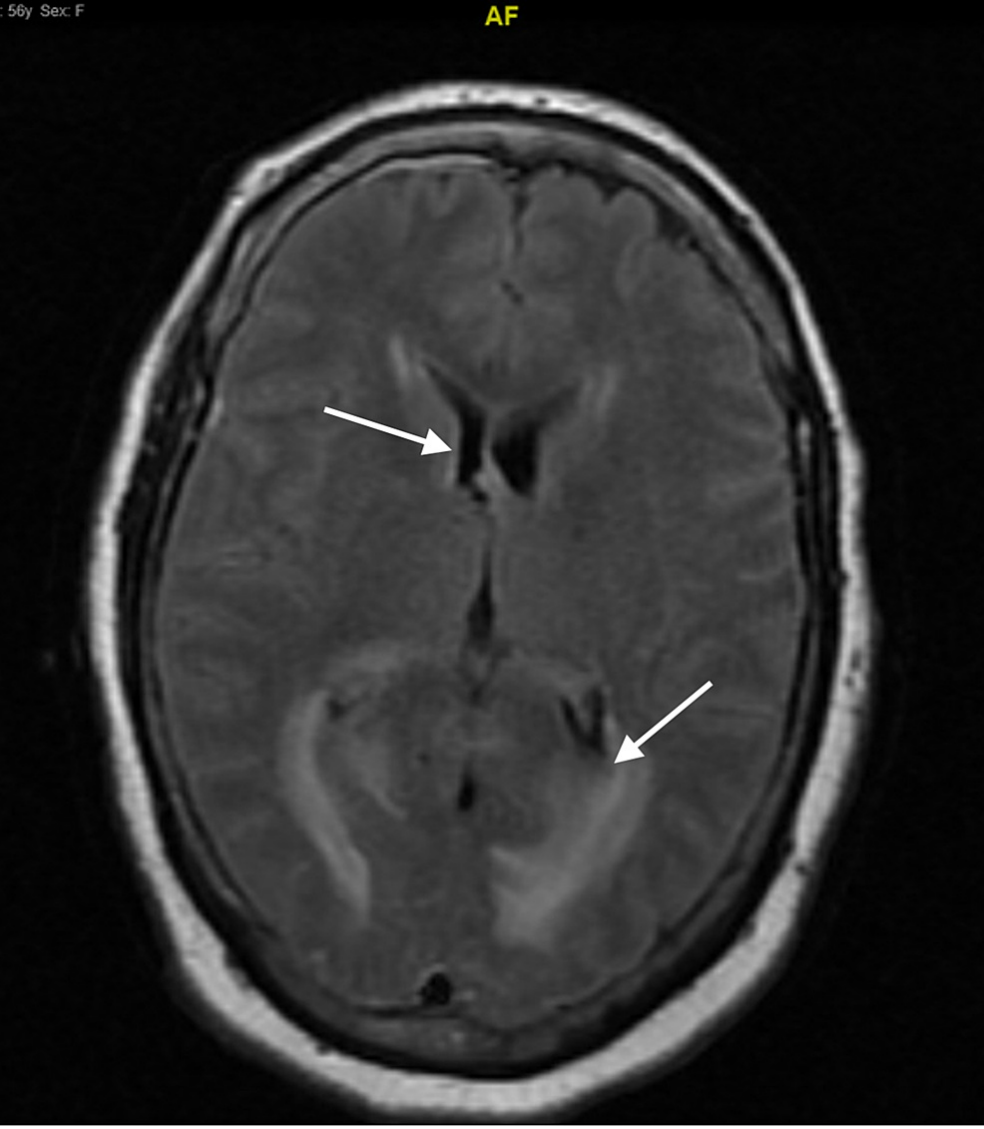
MRI of the brain without contrast in axial view on day 3 of hospitalization The image shows worsening diffuse brain edema with decreased ventricular size.

Repeat CTH after the initial MRI showed diffuse decreased attenuation in the bilateral cerebellum that was causing a mass effect on the fourth ventricle, leading to noncommunicating hydrocephalus. The patient then had a suboccipital craniectomy done on day 2 of hospitalization, with repeat MRI of the brain still showing decreased attenuation in the bilateral cerebellum with mass effect on the fourth ventricle and low lying cerebellar tonsil with upward and downward herniation (Figures 2, 3). She was noted to have no brainstem reflexes. However, repeat head imaging showed improvement of the edema; she slowly regained some reflexes with an overall poor prognosis for further brain recovery. Ultimately, her family proceeded with tracheostomy and percutaneous endoscopic gastrostomy (PEG) tube placement, and the patient was discharged to rehabilitation in hopes of further neurological recovery.

## Discussion

Of the patients that were presented in prior case reports about CHANTER syndrome, most had developed varying degrees of physical and memory impairment but with overall recovery, except for one patient who died [1]. A more recent case of CHANTER syndrome presented a 30-year-old female who recovered almost completely with some mild residual right leg weakness after aggressive medical therapy without surgical decompression [2]. In general, the potential for better outcomes relied on early identification and treatment.

In our case, the initial imaging showed findings consistent with CHANTER syndrome, with subsequent imaging showing the development of obstructive hydrocephalus leading to herniation requiring surgical intervention. However, even with early treatment with osmotic medications and EVD, the patient progressed to needing a decompressive craniectomy. In the end, she still had a poor prognosis with minimal brain function recovery.

Some relevant unknowns that could explain her deterioration include the type and quantity of her substance use and the number of years of substance use. There may be chronic underlying baseline changes or effects on the brain that the substance use had caused, which could have contributed to the progression of her clinical course with CHANTER syndrome versus the progression of other known cases. There has been a case report demonstrating effects on brain areas other than the cerebellum and the hippocampi regions related to CHANTER syndrome, indicating that there is some variability in this syndrome across patients [3]. Another possible explanation could be differences across chronic medical conditions for patients. Some other cases of CHANTER syndrome reported occurred in younger individuals who had relatively little to no chronic problems. In our patient, she was a middle-aged woman with multiple chronic medical conditions that may have impacted the process of her recovery. Nevertheless, based on past case studies of CHANTER syndrome, this patient’s deterioration despite her rapid diagnosis and treatment is an anomaly.

## Conclusions

Patients diagnosed with CHANTER syndrome demonstrated mainly positive outcomes when the syndrome was identified early with subsequent intervention. This case report presents a patient in whom CHANTER syndrome was identified quickly and treated, but the overall prognosis of further neurological recovery beyond regaining some brainstem reflexes remained poor. This demonstrates the unpredictability of the progression of this disease process even with early diagnosis and treatment. It also suggests the need for further studies into the confounding factors that could impact disease progression/outcome, including variables such as the substance of use, the duration and quantity of use, and the chronic medical conditions for each patient.
